# Bilateral ovarian metastasis of a Klatskin tumor: A rare case

**DOI:** 10.4274/tjod.40222

**Published:** 2016-12-15

**Authors:** Sefa Kurt, Çağnur Ulukuş, Seher Nazlı Kazaz, İbrahim Astarcıoğlu

**Affiliations:** 1 Dokuz Eylül University Faculty of Medicine, Department of Obstetrics and Gynecology, İzmir, Turkey; 2 Dokuz Eylül University Faculty of Medicine, Department of Pathology, İzmir, Turkey; 3 Dokuz Eylül University Faculty of Medicine, Department of Internal Medicine, Division of Medical Oncology, İzmir, Turkey; 4 Dokuz Eylül University Faculty of Medicine, Department of General Surgery, İzmir, Turkey

**Keywords:** Klatskin tumor, metastatic ovarian tumor, cholangiocellular carcinoma

## Abstract

Metastatic carcinomas of the ovary have an important place in all ovarian cancers and tumors. They can originate from many organs and systems and may metastasize to the ovary. The most common primary origin of metastasis is the gastrointestinal tract and then breast tissue. Cholangiocellular carcinomas involving the junction of the right and left bile ducts are called Klatskin tumors, and their metastases to the ovaries are very rare. A woman aged 54 years who had been treated previously for Klatskin tumor was admitted to our clinic due to bilateral ovarian masses and high serum calcium 19-9 levels. The preoperative approach, operative, and postoperative management of Klatskin tumor is presented.

## INTRODUCTION

Cholangiocellular carcinomas are rare adenocarcinoma tumors of the bile ducts that may arise anywhere along the biliary tree. Cholangiocellular carcinomas involving the junction of the right and left bile ducts are called Klatskin tumors^(1)^. Ovarian metastases of these tumors are very rare clinical presentations^(2)^. We report bilateral ovarian metastases of a Klatskin tumor because of its rarity, aggressive structure, and difficulties in its management.

## CASE REPORT

**Figure 1:**
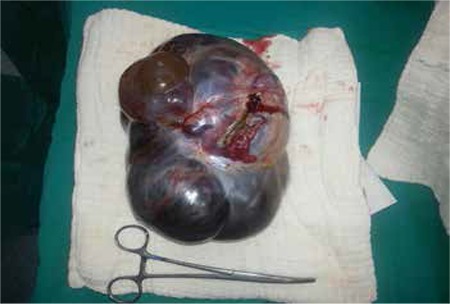
A lobulated metastatic mass originated from the left adnexa, measuring 18x20 cm

**Figure 2:**
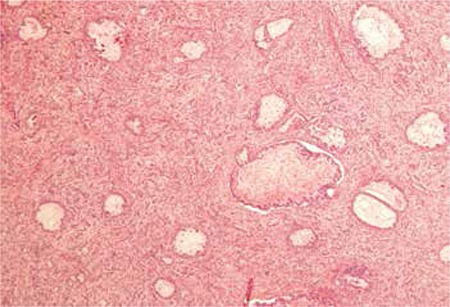
Mucinous adenocarcinoma. Atypical tumor cells containing mucin formed adenoid structures. Also, the stroma between tumor cells and the lumen of mucin formed adenoid structures is shown (hematoxylin&eosin, x100)

A gravida 4, parity two woman aged 54 years who was three years menopausal underwent surgery for cholangiocellular cancer, type B, one year ago (T3BN0, Stage 3B). She received gemcitabine and 5-fluorouracil infusion therapy every two weeks for 8 cycles and chemoradiation with 5-fluorouracil infusions after surgery. After one year, she was admitted to the emergency unit with the main symptoms of abdominal swelling and pain. An abdominal ultrasound scan revealed bilateral ovarian masses and she was referred to our gynecologic oncology section of the department of obstetrics and gynecology. The gynecologic examination of the external genitalia was compatibly normal regarding her age. Bimanual examination revealed a mass that filled the pelvis and extended to the umbilicus. Magnetic resonance imaging showed a cystic, hemorrhagic mass with polypoid protrusions from the cyst wall, measuring 105x95x65 mm, originating from the left ovary. There was also a tumor on the right ovary sized 3.5x4x5 cm, which shared the same properties. Hematologic and biochemical tests were normal. Serum tumor markers calcium (CA) 125 and CA 19-9 were 35.8 U/mL and 1782.7 U/mL, respectively. Upper and lower gastrointestinal system endoscopies were also found normal. She underwent a diagnostic laparotomy. A lobulated 18x20 cm mass that originated from the left ovary and filled the pelvis, and a 5x6-cm cystic septal lesion originating from the right ovary were observed in the lower abdomen (Figure 1). No tumor formation or implant was diagnosed in the pelvic peritoneum or in the upper abdomen. Peritoneal wash fluid and multiple peritoneal biopsies were taken from all quadrants. The left adnexal mass was excised and sent for frozen section examination. The result of frozen section examination was reported as a malignant tumor with mucinous features. Debulking surgery was performed. Microscopic examination of paraffin sections from bilateral ovaries and omentum revealed mucinous adenocarcinoma (Figure 2). Malignant cells were also observed in the peritoneal wash fluid. A pathologic evaluation was also performed by comparing the findings of the previous material diagnosed as Klatskin tumor with the wash fluid sample and it was observed that both materials had similar characteristics of the same tumor. Immunohistochemical marker tests were performed to determine the tumor’s primary origin. There was positive staining for CDX2, cytokeratin 19, and cytokeratin 20 detected in the tumor cells. Cytokeratin 7, PAX8, estrogen, and progesterone receptors were found negative. The immunohistochemical findings supported pancreaticobiliary and gastrointestinal origin. The final result of the pathologic examination confirmed a metastatic adenocarcinoma of the hepatic ducts.

## DISCUSSION

Ovarian cancer remains an important place among gynecologic cancers because of the high mortality rate. The ovaries are also the target organs for metastasis from many malignancies. Metastatic cancers of the ovary constitute 5-10% of total ovarian tumors, and 10-30%’s of total malignant ovarian tumors^(2)^. The incidence of metastatic ovarian tumors is increasing and the rates vary by countries. Frequency of metastatic ovarian cancer in Japan is reaching up to 40% because of high incidence of gastric cancers. However it is decreasing to 3% in Uganda^(3)^. In addition, the immunohistochemical markers which are used to diagnose the primary origin also contribute to increasing incidence^(2)^.

The differential diagnosis of primary and metastatic tumors may be difficult. The pathological evaluation should be done with clinical evaluation in determining the primary origin. Metastatic ovarian cancer can mimic primary ovarian cancers, so imaging methods may be insufficient. The most common non-genital system tumors which metastasize to the ovary are from gastrointestinal system such as; breast, and hematopoietic systems^(2)^.

The structure of the ovary during the intraoperative observation may help for clinic differential diagnosis. The macroscopic presence of bilateral tumor, the implant on the ovarian surface, extra-ovarian mass and solid cystic components may be a predictor of metastatic ovarian tumors. All the bilateral tumors and unilateral tumors smaller than 10 cm are more likely to be metastatic cancer; and the unilateral tumors larger than 10 cm are more likely to have a primary ovarian origin^(4)^.

A mucinous adenocarcinoma of the ovary can be the primary mucinous adenocarcinoma of the ovary or as in our case can originate from other organs or systems which can develop mucinous adenocarcinoma, including particularly the gastrointestinal tract. Histopathological features including infiltrative and nodular invasion pattern, lymphovascular invasion and the presence of signet ring cells can alert for the metastases^(4)^. In addition, immunohistochemical markers can contribute to determining of the primary origin. However, in the presence of an ovarian tumor with mucinous adenocarcinoma morphology, the similarity in immunohistochemical findings of the primary and metastatic tumors should be kept in mind. Therefore; histopathological features, medical history, clinical and laboratory findings should be evaluated for the discrimination of primary or metastatic tumor. Although there are not any specific tumor markers for cholangiocellular carcinoma, increased levels of CA 19.9 (>100 U/mL) should be significant for pancreatic and cholangiocellular carcinomas^(5)^. Despite the fact that the normal appearance of gastro intestinal system and pancreas, increased levels of CA 19.9 (1782.7 U/mL) is remarkable in our case and appropriate with isolated ovarian metastasis. Besides it has been reported that elevated levels of CA 19.9 is correlated with poor prognosis and advanced stage disease^(6)^. The metastasis rate from the biliary tumors to the ovaries is very low in the current literature^(1)^. The cases diagnosed with ovarian metastasis before the diagnosis of primary cholangiocellular carcinoma would show much worse prognosis^(7)^. Whenever synchronous or metachronous metastases progress, identified surgical resection should be always recommended to these patients^(8)^. In conclusion, metastatic ovarian tumors are a challenging condition for the gynecologists. The difficulties in the differential diagnosis of the metastatic and primary ovarian tumors required a detailed analysis. As many distant organs and tissues may metastasize to the ovaries the patients should be evaluated with medical history, examination of the genital and non-genital system, laboratory findings, as well as intraoperative observation. Prognosis of metastatic ovarian tumors is worse than the primary tumors. Multidisciplinary approach is important in the management of such tumors.

## References

[1] Bhardwaj N, Garcea G, Dennison AR, Maddern GJ (2015). The Surgical Management of Klatskin Tumours: Has Anything Changed in the Last Decade?. World J Surg.

[2] Kondi-Pafiti A, Kairi-Vasilatou E, Iavazzo C, Dastamani C, Bakalianou K, Liapis A, et al (2011). Metastatic neoplasms of the ovaries: a clinicopathological study of 97 cases. Arch Gynecol Obstet.

[3] Bayar Ü, Kayar E (2004). Metastatic Ovarian Tumors. Türkiye Klinikleri J Gynecol Obst.

[4] Jain V, Gupta K, Kudva R, Rodrigues GS (2006). A case of ovarian metastasis of gall bladder carcinoma simulating primary ovarian neoplasm: diagnostic pitfalls and review of literature. Int J Gynecol Cancer.

[5] Koçak E, Koçak G, Beşir FH, Can M (2007). A case of Klatskin Tumor with High Levels of CA 19-9. Turkiye Klinikleri J Med Sci.

[6] Sal V, Demirkiran F, Topuz S, Kahramanoglu I, Yalcin I, Bese T, et al (2016). Surgical Treatment of Metastatic Ovarian Tumors From Extragenital Primary Sites. Int J Gynecol Cancer.

[7] Yeshmine F, Hussain M, Dey S, Rahman T, Khatun M (2013). Ovarian Metastases Caused by Cholangiocarcinoma: A rare Krukenberg’s Tumor: A Case Study. Bangladesh J of Radiol.

[8] Rosa F, Marrelli D, Morgagni P, Cipollari C, Vittimberga G, Framarini M, et al (2016). Krukenberg Tumors of Gastric Origin: The Rationale of Surgical Resection and Perioperative Treatments in a Multicenter Western Experience. World J Surg.

